# Morphological Traits Shape Foraging Scale but Not Precision: Divergent Responses of Four Tree Species to Water and Nutrient Heterogeneity

**DOI:** 10.3390/plants15070998

**Published:** 2026-03-24

**Authors:** Liuduan Wei, Tianxin Dong, Liufeng Lan, Jian Lin, Xianwen Li, Miao Yu, Chengyang Xu

**Affiliations:** 1 Research Center for Urban Forestry of Beijing Forestry University, Key Laboratory for Silviculture and Forest Conservation of the Ministry of Education, Beijing 100083, China; 18376684420@163.com (L.W.); dongtianxin0622@163.com (T.D.); 15600990834@163.com (X.L.); 2 Guangxi State-Owned Huangmian Forest Farm, Liuzhou 545618, China; xinchc73@163.com; 3 Nanning Arboretum, Guangxi Zhuang Autonomous Region, Nanning 530033, China; 18275882271@163.com

**Keywords:** fine-root traits, root foraging strategies, resource heterogeneity, patch types, species specificity, nutrient availability

## Abstract

Soil nutrients and water are often distributed heterogeneously in space, yet how plant roots forage in response to such heterogeneity and how their strategies relate to functional traits remain poorly understood. Here, we conducted an indoor pot experiment manipulating water and nutrient supply in both homogeneous and heterogeneous patch patterns using seedlings of four tree species, focusing on root functional traits and foraging strategies. The results indicate that root foraging behavior exhibits both resource specificity and species specificity: roots tend to proliferate toward nutrient-rich and low-water patches as an adaptive strategy. Although no strict dichotomy was observed between high foraging scale (low precision) and low foraging scale (high precision) strategies under heterogeneous conditions, fine-rooted species (*Acer truncatum* and *Koelreuteria paniculata*) exhibited traits leaning toward “precise foraging”, whereas coarse-rooted species (*Prunus davidiana* and *Quercus variabilis*) tended toward a conservative “random walk” pattern, with no trade-off between root foraging scale and precision. Root morphological traits exerted significant nonlinear regulation on foraging scale: root biomass foraging scale (FS_RB_) correlated positively with root diameter (RD) but negatively with specific root length (SRL) and specific root area (SRA); root length foraging scale (FS_RL_) correlated positively with root length (RL), root tip number (RTN), SRL, and SRA. In contrast, root morphological traits could not explain the variation in foraging precision, suggesting that foraging precision constitutes another distinct dimension in root-trait space. In summary, this study provides key insights into the foraging strategies of plant roots in heterogeneous environments, expanding our understanding of the multidimensionality of root functional traits.

## 1. Introduction

Soil resources, particularly relatively immobile elements such as nutrients and water, are typically distributed heterogeneously across space and time [1,2,3,4]. Roots, as one of the two main resource-acquiring organs in plants, are responsible for capturing water and soil-borne nutrients. Hence, plant survival and success critically depend on the ability of fine roots to effectively locate and take up these essential resources. The spatial arrangement of fine roots, which varies considerably along both vertical and horizontal gradients, is shaped by a combination of geometric/topological constraints and functional adaptations, together defining root foraging strategies [5,6]. Therefore, the spatial matching between soil resource availability and fine-root distribution is a fundamental determinant of successful nutrient and water acquisition in plants.

Root systems of many plant species possess pronounced phenotypic plasticity to respond to their environment [7]. This plasticity underpins a key foraging response that enhances a plant’s capacity to exploit heterogeneous resource distributions [8,9,10], a process commonly termed root foraging. Root foraging ability, which reflects the efficiency of resource capture, can be described by foraging-scale and -precision in competition by plants for resources [11]. Root foraging scale quantifies nutrient capture to be monopolized by an intact root system [7], while root foraging precision quantifies the tendency to proliferate roots in resource-rich patches [10]. Both scale and precision are widely used in split-root experiments to assess different dimensions of root foraging behavior across diverse species and environmental contexts [12,13,14].

The proliferation mechanisms are based mainly on root biomass allocation and a series of morphological changes in roots [15,16,17]. Specifically, functional traits serve as the mechanistic link between root architecture and foraging performance [14,16]. Foraging scale is enhanced primarily through root elongation and the consequent increase in total root length, surface area, and root biomass, allowing plants to explore larger soil volumes, effectively increasing the probability of encountering spatially distinct resource patches [18]. Conversely, foraging precision is achieved by developing extensive lateral branching and complex topological structures. High branching intensity facilitates the intensive occupation of local soil zones [13], markedly improving precision through the rapid concentration of absorptive fine roots within enriched patches. A trade-off between foraging scale and precision has been reported in some studies [11,19,20], whereas others indicate that the relationship between these parameters is inconsistent or context-dependent [14,21,22,23,24]. Despite extensive research on the scale–precision relationship [12,14,20,24,25], there are only a few comparative studies linking root traits to root foraging precision and scale [16,17,26], and robust empirical testing of these linkages is lacking, especially in co-existing species.

The size and mode of root morphological plasticity of plants in heterogeneous soil strongly depend upon the plants themselves [27]. The differences in root foraging ability have been thought to be connected to plant growth rates and acquisitive strategies [12]. Previous work has shown that interspecific variation in fine-root diameter influences plant responses to nutritionally heterogeneous environments [28,29,30]. Thin-rooted species are generally considered to possess higher specific root length [31] and root growth rate [28], and even greater root plasticity or foraging precision compared to thick-rooted species [30]. However, some studies report similar foraging precision between thin- and thick-rooted species [26], raising questions about whether foraging strategies truly diverge according to species-specific root traits. While the ‘foraging scale’ is often regarded as an inherent genotypic trait, making ‘foraging precision’ largely species-dependent [13], environmental heterogeneity does exert a notable influence, allowing foraging precision to respond to soil conditions when root system size varies [32,33]. Indeed, this regulation is resource-specific, especially when plant species adopt different strategies to obtain different resources. For instance, Chinese fir root length density decreased with phosphorus addition but remained unchanged with nitrogen addition [34]. Similarly, although drought has been shown to expand root foraging range [7], other evidence indicates that roots preferentially proliferate in water-rich patches [10]. Given that soil water and nutrients are both essential [35] and often heterogeneously distributed resources [3,10,17], understanding these divergent responses is vital. However, few studies have simultaneously examined water and nutrient patches in the same experimental system. Consequently, despite evidence of resource-specific root responses, comparative insights into how a single plant species simultaneously adjusts its roots to water versus nutrient heterogeneity remain scarce.

*Koelreuteria paniculata*, *Quercus variabilis*, *Acer truncatum*, and *Prunus davidiana* frequently coexist in mixed forests across northern China, a region characterized by arid and semi-arid climates with nutrient-poor soils [36]. These species exhibit a spectrum of root architectural strategies, ranging from coarse- to fine-rooted types, and differ in their growth rates. Consequently, the four species likely employ distinct foraging strategies to adapt to heterogeneous soil, and such differentiation could enhance local species coexistence and soil-resource-use efficiency [27,37]. We established both homogeneous and heterogeneous supply regimes for water and nutrient patches. Our main aim was to quantify the scale and precision of root foraging in these species under varying resource conditions. We tested the following hypotheses: (1) Based on previous evidence of root proliferation in resource-rich zones, we hypothesized that roots would consistently proliferate in both water- and nutrient-enriched patches. (2) The root foraging strategies are species-specific modified by their inherent differences in root morphology. (3) There is a trade-off between root foraging scale and precision, and that both foraging parameters are significantly correlated with fine-root morphological traits in heterogeneous water and nutrient environments.

## 2. Results

### 2.1. Effects of Resource-Patch Treatments on Root Foraging Scale

**Figure 1:**
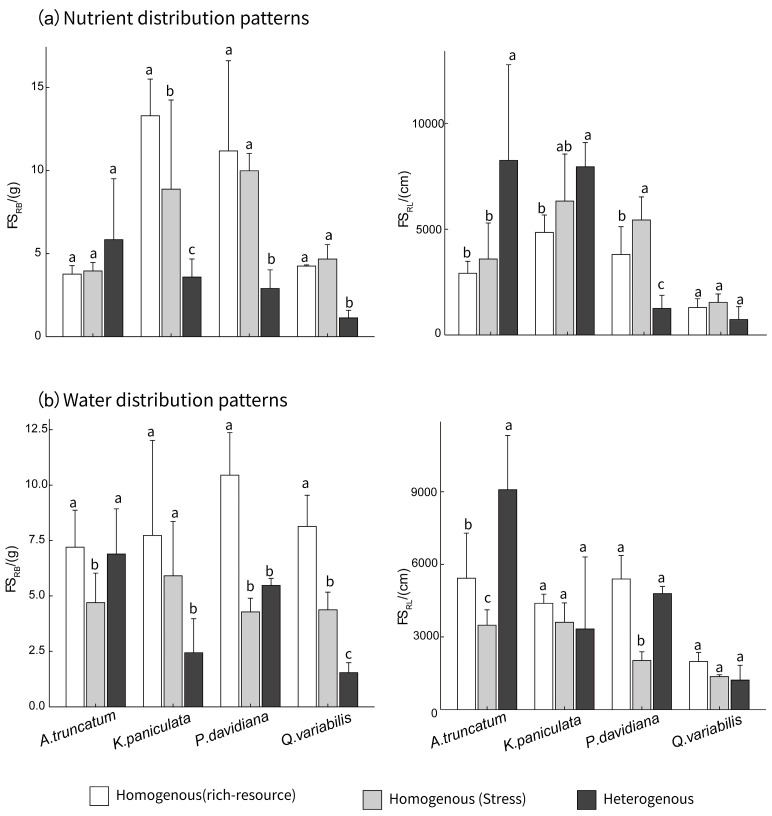
Root foraging scale in four tree species seedlings under (**a**) nutrient and (**b**) water distribution patterns. FS_RB_, root biomass foraging scale; FS_RL_, root length foraging scale. Means are presented as columns; error bars represent standard errors. Different letters above bars indicate significant differences among the treatments for each tree species separately.

Significant interactive effects of resource-patch treatments and tree species were observed on root biomass foraging scale (FS_RB_, *F* = 35.04, *p* < 0.001) and root length foraging scale (FS_RL_, *F* = 23.48, *p* < 0.001; Table S2). Under homogeneous patterns, water stress reduced both FS_RB_ and FS_RL_ compared to water-rich conditions, with *P. davidiana* and *Q. variabilis* showing the most pronounced reductions (Figure 1). In contrast, under homogeneous nutrient stress, FS_RB_ showed species-specific responses: it increased in *K. paniculata* and *P. davidiana* but decreased in *A. truncatum* and *Q. variabilis*. For FS_RL_, all four species showed an increase under nutrient stress. In heterogeneous environments, *A. truncatum* displayed the strongest phenotypic plasticity, with both FS_RB_ and FS_RL_ significantly higher than under homogeneous conditions. *K. paniculata* showed increased FS_RL_ but decreased FS_RB_ under nutrient heterogeneity relative to homogeneous nutrient supply. Conversely, *P. davidiana* and *Q. variabilis* were inhibited in heterogeneous settings, particularly under nutrient heterogeneity. *Q. variabilis* exhibited the lowest root foraging scale among all species under both drought and nutrient heterogeneity. Its FS_RB_ was markedly lower in heterogeneous treatments compared to homogeneous treatments; although the difference in FS_RL_ was not statistically significant, it was numerically lower in the heterogeneous environment (Figure 1).

### 2.2. Effects of Resource-Patch Treatments on Root Foraging Precision

**Figure 2:**
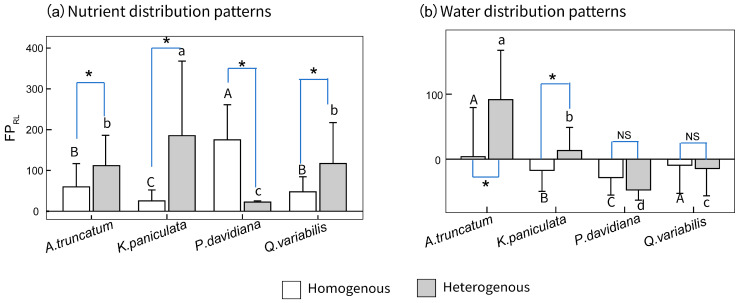
Root foraging precision (FP_RL_) under heterogeneous vs. homogeneous stresses. (**a**) Nutrient distribution patterns. (**b**) Water distribution patterns. Values of root foraging precision above 1 mean that plants created more roots in the resource-rich area; values below 1 mean that plants created more roots in the resource-poor area. Capital letters indicate significant differences among species under heterogeneous conditions, and lowercase letters indicate differences under homogeneous conditions. Asterisks (*) indicate significant differences between heterogeneous and homogeneous conditions for the same species, and NS indicates no significant difference.

A significant interaction indicated that the effect of resource-patch treatments on root foraging precision differed among tree species (*p* < 0.001; Table S3). Under both homogeneous and heterogeneous nutrient environments, all four species exhibited positive foraging precision (FP_RL_ > 0). The response to nutrient heterogeneity was species-specific:, *A. truncatum*, *K. paniculata*, and *Q. variabilis* showed higher foraging precision under heterogeneous nutrient conditions, whereas *P. davidiana* displayed greater precision in homogeneous nutrient settings (Figure 2). In water-patch treatments, species-specific responses in root sensitivity were also pronounced. Roots of *A. truncatum* and *K. paniculata* tended to proliferate in water-rich patches, while those of *P. davidiana* and *Q. variabilis* showed a tendency to extend toward water-stressed patches. Notably, under heterogeneous resource-patch, the root foraging precision of *A. truncatum* and *K. paniculata* was significantly higher than that of *P. davidiana* and *Q. variabilis*.

**Figure 3:**
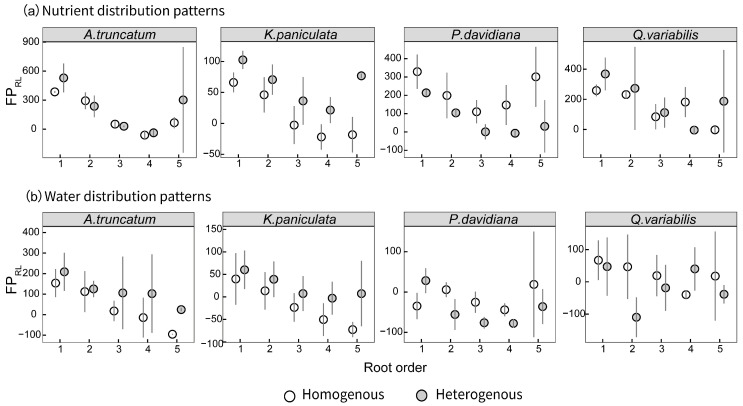
Root foraging precision (FP_RL_) across root orders under heterogeneous and homogeneous stresses for (**a**) nutrient and (**b**) water distribution patterns.

Differences in foraging traits across root orders further highlighted functional differentiation within fine-root systems (Figure 3). Lower-order roots (1st–2nd order) tended to exhibit higher FP_RL_ under nutrient heterogeneity compared to higher-order roots (4th–5th order), and this enhancement of sensitivity in heterogeneous environments was more pronounced in lower-order roots. In contrast, under water stress, the foraging precision of lower-order roots was suppressed, whereas higher-order roots maintained relatively stable responses.

### 2.3. Relationships Between Root Traits and Root Foraging Traits

**Figure 4:**
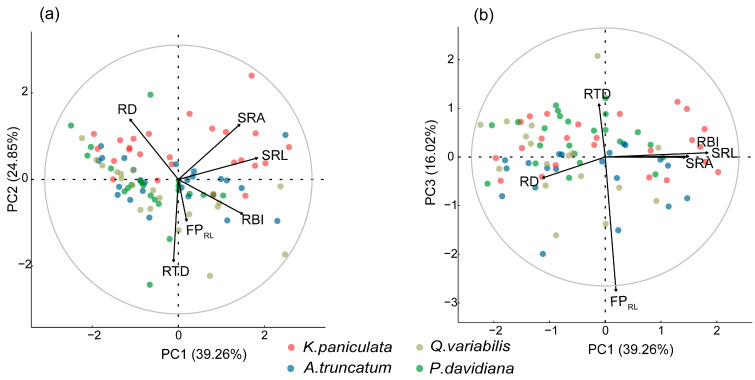
Principal component analysis of root foraging precision and root traits for four tree species seedlings, showing the relationships between PC1 and PC2 (**a**), and PC1 and PC3 (**b**). RD, root diameter; SRL, specific root length; SRA, specific root area; RTD, root tissue density; RBI, root branching intensity; FP_RL_, root length foraging precision.

We did not find any individual traits to be significant predictors of root foraging precision (*p* > 0.05), regardless of nutrient or water distribution patterns (Table S4). Root traits and foraging precision were collectively represented by three principal component axes that captured 80% of the total trait variation. Root foraging precision was primarily associated with the third axis (loading = −0.90), while its contribution to the first two axes was comparatively limited (Figure 4, Table S5).

**Figure 5:**
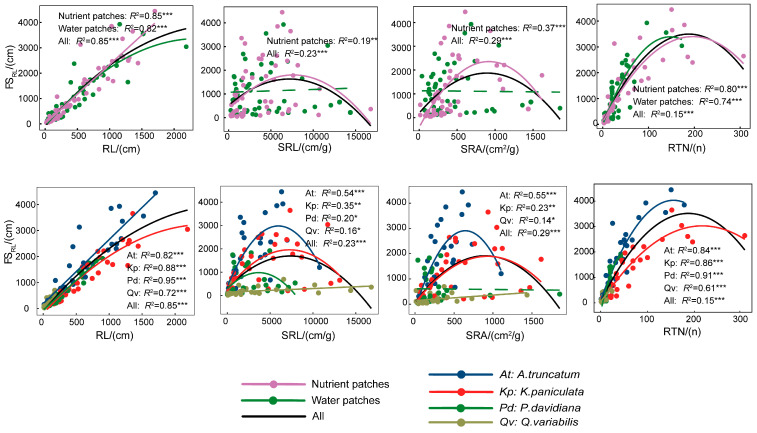
Relationship between root length foraging scale and root system traits. RL, root length; SRL, specific root length; SRA, specific root area; RTN, root tip number; FS_RL_, root length foraging scale. *R*^2^ denotes coefficients of determination from linear regression. Solid lines represent significant relationships (*p* < 0.05) and dashed lines represent no significant relationships (*p* > 0.05); *, ** and *** indicate significance at *p* < 0.05, *p* < 0.01 and *p* < 0.001 level, respectively.

**Figure 6:**
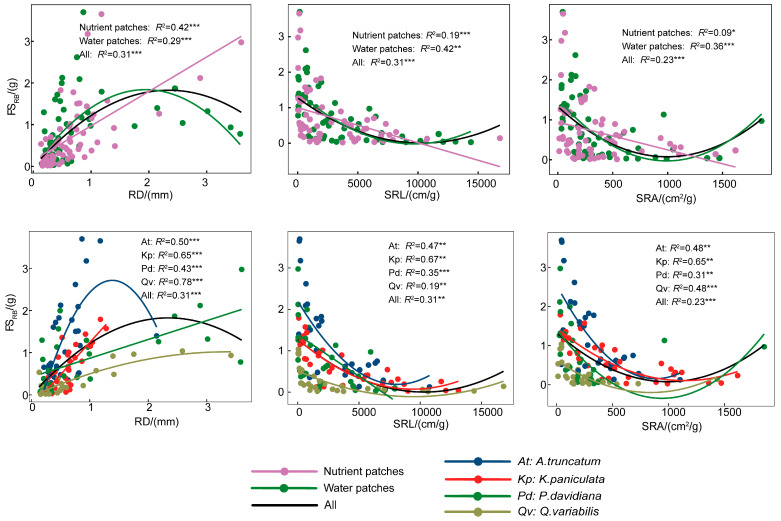
Relationship between root biomass foraging scale and root system traits. RD, root diameter; SRL, specific root length; SRA, specific root area; FS_RB_, root biomass foraging scale. *R*^2^ denotes coefficients of determination from linear regression. Solid lines represent significant relationships (*p* < 0.05); *, ** and *** indicate significance at *p* < 0.05, *p* < 0.01 and *p* < 0.001 level, respectively.

In contrast, root foraging scale was significantly correlated with several root traits (Table S4). FS_RB_ correlated positively with RD but negatively with SRL and SRA. FS_RL_ correlated positively with RL, RTN, SRL, and SRA (Table S4). Furthermore, the relationships between root traits and foraging scale differed distinctly across resource-patch types (Figure 5 and Figure 6). In nutrient patches, FS_RL_ exhibited stronger associations with root traits, particularly SRL and SRA. Additionally, the relationship for FS_RB_ was best fitted by a linear model under nutrient heterogeneity but followed a quadratic regression under water-patch heterogeneity. Further regression analysis revealed nonlinear relationships between root foraging scale and root traits. FS_RL_ displayed an inverted U-shaped response to increases in RL, RTN, SRL, and SRA, a trend that was particularly strong in *A. truncatum* and *K. paniculata* (Figure 5). Conversely, FS_RB_ showed a U-shaped relationship with SRL and SRA, a pattern consistent across all studied species (Figure 6). FS_RB_ also displayed an inverted U-shaped relationship with RD, which was most pronounced in *A. truncatum* (*R*^2^ = 0.50, *p* < 0.001; Figure 6).

### 2.4. Relationship Between Root Foraging Scale and Precision

**Figure 7:**
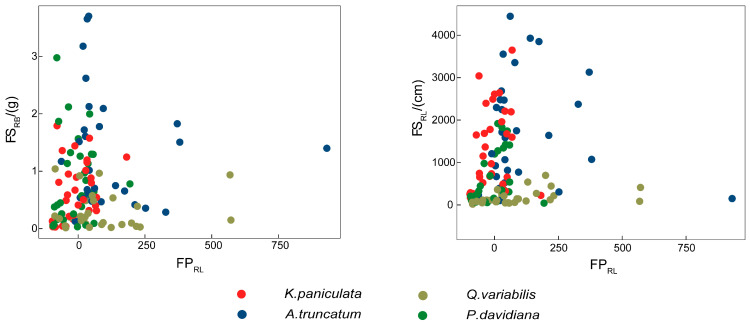
A lack of relationship between foraging precision and foraging scale of roots across 4 tree species in resource patches. FS_RB_, root biomass foraging scale; FS_RL_, root length foraging scale; FP_RL,_ root length foraging precision.

In both water- and nutrient-patch treatments, Pearson correlation coefficients between foraging-scale metrics (FS_RB_, FS_RL_) and FP_RL_ did not reach statistical significance (*p* > 0.05; Table S6). All four tree species consistently showed no significant association between foraging precision and scale (Figure 7).

## 3. Discussion

### 3.1. Effect of Patch Types on Root Responses to Resource Heterogeneity

Our analysis indicated that resource type substantially influenced both root foraging precision and scale under heterogeneous conditions. When interpreting the observed response, the context in which the response had been expressed (e.g., attributes of the patch) was as important as the actual response itself [2]. Given the response-specific responses are ubiquitous [38], testing whether plant responses to response patches are phylogenetically and taxonomically conserved across species should be done under the same set of response environments [39]. *K. paniculata*, *P. davidiana*, and *Q. variabilis* responded to nutrient stress by increasing root length in nutrient-enriched patches, *A. truncatum* exhibited positive responses to both water- and nutrient-enriched patches, but its response to nutrient patches showed greater variability (Figure 2). These results indicate that root sensitivity to nutrients was significantly stronger than to water among the four tree species. The capacity of roots to proliferate in resource-enriched patches was determined by the plant’s specific resource requirements, sensitivity, and tolerance [27,40,41].

Soil nutrient availability is inherently heterogeneous at various spatial scales, forming a mosaic in which nutrient supply varies from point to point [42]. In heterogeneous nutrient patches, root sensitivity and foraging precision were higher than in homogeneous environments, with proliferation directed toward nutrient-rich patches. As a critical limiting factor for plant growth, nutrient-rich patches in heterogeneous settings provide more abundant nutrient supply, and plants exhibit root plasticity (e.g., increased root length, adjusted branching) to allocate more roots to nutrient-rich areas, thereby maximizing nutrient uptake efficiency [9,43]. Furthermore, the relationship between foraging scale and root traits is particularly pronounced in nutrient patches, likely because the heterogeneity of nutrient distribution more strongly drives adaptive differentiation in root morphology [9,17]. For example, fine root traits such as high specific root length and abundant root tips can more effectively translate into advantages in spatial exploration within nutrient patches [44]. In contrast, in water patches, the physical processes of water movement and their physiological stress effects on roots [45,46] may partially obscure the direct correlation between traits and foraging scale. In both heterogeneous and homogeneous water environments, root sensitivity and foraging precision are negative (i.e., roots proliferate toward low-water patches), which relates to the physiological properties of water and species-specific adaptations: excessive water may lead to root hypoxia, root rot, or other stresses [47,48], while low-water patches, despite lower moisture, may offer better soil aeration. This response was particularly evident in the coarse-rooted species *P. davidiana* and *Q. variabilis*, which showed a tendency to extend toward water-stressed patches (Figure 2). Additionally, the studied tree species exhibit strong drought tolerance, making low-water patches more aligned with their physiological demands. Consequently, in heterogeneous water environments, roots tend to proliferate toward low-water patches, resulting in negative sensitivity and foraging precision. This differential response reflects a trade-off in plant adaptation to water availability—water-rich patches are not always the optimal choice, and proliferation toward low-water patches represents a long-term adaptive outcome of species to water availability.

### 3.2. Diverse Root Foraging Strategies Among Tree Species

When local soil resources are supplied in patches, root systems of different species exhibit distinct responses [1,14,41,49]. Our study quantified a wide range of plasticity in root traits in response to resource-rich patches, indicating that co-existing tree species employ divergent foraging strategies to enhance water and nutrient acquisition. This supports the first hypothesis 2. Different tree species may exploit distinct soil resource pools through differentiated root foraging traits [17], with these differences closely linked to their root characteristics. Plant root systems display considerable plasticity in soil spatial distribution, a behavior captured by two key dimensions: foraging scale (spatial extent of exploration) and foraging precision (degree of localized proliferation) [12,50]. Although roots have been suggested to follow a “random walk” strategy due to their inability to predict nutrient distribution [51], other studies have demonstrated their capacity for precision foraging through localized proliferation responses to soil heterogeneity [4,9]. This study found no significant trade-off between root foraging scale and precision, suggesting that tree species can occupy varied positions along a “precise foraging–random walk” continuum, enabling differentiated resource acquisition through distinct trait combinations.

*A. truncatum* demonstrated high foraging precision, indicating a clear tendency toward a “precise foraging” strategy. In heterogeneous environments, this species exhibited “high-efficiency precise” characteristics, maintaining both high foraging scale and precision. Its well-developed root morphological plasticity, such as high specific root length and root branching intensity, allows for the rapid formation of high-surface-area absorptive networks upon detecting resource patches [1]. Notably, while its FS_RB_ was relatively low, its high FS_RL_ enabled more efficient spatial exploration per unit carbon invested [52]. FS_RL_ may be a better indicator of competitiveness than FS_RB_ [49], though higher FS_RL_ is often associated with faster root turnover, potentially accelerating local carbon and nutrient cycling [53]. *K. paniculata* also showed high foraging precision, yet its strategy leans more toward “economically precise foraging”. Similarly to *A. truncatum*, it responded strongly to nutrient heterogeneity by proliferating in nutrient-rich patches. However, its response to water heterogeneity was less pronounced (Figure 2). This species significantly increased root length at a lower biomass cost to occupy broader soil volumes while precisely targeting resource-rich patches [27], demonstrating an efficient foraging strategy that is particularly effective in nutrient-patch environments. In contrast, *P. davidiana* and *Q. variabilis* exhibited more conservative responses with lower precision, resembling a “random walk” pattern. These species showed limited plasticity in their foraging precision in response to heterogeneity. Thick-root species appear to have limited capacity to forage in the heterogeneous soil [26], yet they can persist in stable patches through longer root lifespan [54]. Although their foraging precision and scale are relatively low in heterogeneous settings, increasing construction costs in resource-rich patches may reduce excessive branching and intra-root competition, supporting long-term root development [4,55]. By constructing thicker root structures, plants can sustain survival and growth in resource-unstable or highly stressful habitats [56].

Overall, fine-rooted species tend to adopt precise foraging strategies, whereas coarse-rooted species are more likely to expand their foraging scale and employ “random-walk” strategies, reflecting an ecological trade-off in resource acquisition via contrasting construction strategies. The diversity in belowground response to water and nutrients that we measured contrasts with the relative constancy in the direction of aboveground responses to nitrogen shortage measured in nine herbaceous species by Freschet et al. [57]. Also, Kramer-Walter et al. [31] showed that the root traits are multidimensional in contrast to the leaf traits, suggesting that plants may have different adaptations to resource limitations belowground. Together, the integration of resource-specific and species-specific responses can generate diverse foraging strategies [41], potentially underpinning species coexistence [26]. This underscores that trait responses to specific resources vary across species, and extrapolating findings to other species, traits, or resources should be approached cautiously.

### 3.3. Root Traits Affect Foraging Scale but Not Foraging Precision

Although changes in root traits have been proposed to influence foraging capacity, such as thin root diameter, high specific root length and root tips are associated with high root foraging precision. However, based on our analysis and the published studies, we agree with that there may be no simple and definitive “rule” for explaining the variation in root and root foraging across species [39]. Consistent with emerging hypotheses [14,16], the foraging precision of the four tree species studied here showed no association with any measured root morphological traits. As root foraging precision did not follow any previously proposed gradient patterns in the nutrient acquisition trait space of trees, we suggest that foraging precision constitutes another distinct dimension in root trait space [16]. This likely arises because precision is more directly governed by physiological and molecular processes, such as signal perception, hormonal regulation, and mycorrhizal symbiosis [4,16,39], rather than by morphology alone. This supports the view proposed by Robinson (2005) that root responses to nutrient variations should be understood as an integrated system operating on multiple organizational levels, extending beyond mere morphological adjustments [42].

In contrast to foraging precision, root foraging scale showed significant correlations with root traits, indicating that a plant’s spatial exploration capacity is strongly constrained by its root architecture and construction costs. The positive correlation between FS_RB_ and RD, together with negative correlations with SRL and SRA, suggests that biomass accumulation is primarily linked to the extension of higher-order roots (Figure S1). This reflects a “thick-walled” strategy requiring higher biomass investment to construct sturdy, low-surface-area roots, which may enhance persistence and transport but limit soil exploration per unit biomass [58]. Conversely, the positive correlations between FS_RL_ and RL, RTN, SRL, and SRA indicate that a higher FS_RL_ is associated with the proliferation of lower-order roots (Figure S1). A “fine and dense” root architecture characterized by high RL and RTN can effectively expand the root–soil interface, enhance spatial exploration, and improve detection and utilization of heterogeneous patches [36]. The association between FS_RL_ and root traits is particularly pronounced in fine-rooted tree species, such as *A. truncatum* and *K. paniculata*. Notably, the U-shaped (or inverted U-shaped) nonlinear relationships revealed by regression analysis further deepen our understanding of trait–function associations. The nonlinear patterns between FS_RL_, FS_RB_, and root traits indicate that foraging scale does not increase monotonically with trait values but reaches an optimum within a specific range, reflecting multiple trade-offs between structure and function. When trait values (such as high SRL, high SRA, or high RTN) exceed a certain threshold, they may enhance short-term resource exploration capacity [59], but also reduce structural support, disrupt carbon allocation, or increase physiological maintenance costs [60,61,62,63], thereby lowering overall foraging efficiency. For example, FS_RL_ peaked at moderate trait values but declined when values were excessively high due to greater structural vulnerability and metabolic burden. Similarly, FS_RB_ performed optimally within specific trait combinations, and configurations that were either too high or too low in root traits can diminish the efficiency of biomass-based spatial exploration. These nonlinear relationships collectively indicate that root foraging behavior is regulated by complex additive, synergistic, or antagonistic interactions among traits [64], with efficiency maximized through a dynamic balance of structural support, resource exploration, and metabolic cost. The nonlinearity also implies that, under given environmental conditions, different trait combinations can achieve similar foraging efficiency, providing a new perspective for explaining belowground niche differentiation and species coexistence. These results underscore the role of root traits as key indicators of plant belowground strategies.

### 3.4. Research Limitations

Although this study elucidates the foraging behavior of different tree species in water and nutrient patches, uncertainties and gaps remain due to constraints in experimental design, while also providing valuable insights for future research. First, the limited number of tree species and exclusion of mycorrhizal foraging constrain deeper insights. Different mycorrhizal types (e.g., arbuscular vs. ectomycorrhizal) show distinct foraging behaviors, as symbionts modify resource acquisition through hyphal networks or root physiology [4,44]. Expanding the range of species with diverse mycorrhizal associations would help clarify how root–mycorrhizal systems explore nutrient and water patches and whether mycorrhizal dependency influences trade-offs between foraging scale and precision. Second, the nutrient heterogeneity in this study was created by manipulating the soil-to-sand ratio, which was considered merely variation in the overall soil nutrient pool, without distinguishing between specific nutrient deficiencies (e.g., N, P, or K limitation). Since plant growth and root proliferation are regulated by the law of minimum and roots respond specifically to different nutrient ions [2], future studies manipulating individual elemental forms would further elucidate the distinct foraging mechanisms for specific resources. Third, the inherent constraints of the pot experiment must be acknowledged. The restricted rooting volume in pots may artificially intensify competitive pressure and exaggerate the influence of water and nutrient patches due to the limited growing space for roots to explore compared to natural field conditions. Fourth, our experimental design focused on single-resource heterogeneity (either water or nutrients) and did not test the interactive effects of co-occurring water and nutrient stress. While this approach allowed us to isolate foraging responses to individual resources, a full factorial design would be necessary to understand how plants prioritize and forage for multiple, simultaneously limiting resources. Taken together, great future efforts are needed to understand how mycorrhizal symbiosis, specific nutrient limitations, and field environments modulate root foraging strategies across diverse tree species in heterogeneous resource landscapes.

## 4. Material and Methods

### 4.1. Site Description

From early March to mid-October 2024, a pot experiment was conducted in a simple greenhouse located at the Puzhaoyuan Nursery, which is part of the Miaofengshan Experimental Forest Farm, Beijing Forestry University, Beijing, China. The nursery is situated at 40°3′45″ N, 116°6′55″ E, with an elevation of 130 m above sea level. The site experiences a temperate continental climate, with a mean temperature of 12.1 °C, annual precipitation of 644 mm, and annual sunshine duration of 2769 h. Throughout the experiment, mean greenhouse temperature was 25.6 °C (range: 16.6–35.2 °C) and mean relative humidity was 60% (range: 50–75%). The greenhouse (dimensions: 10 m × 4 m × 3 m) was fitted with roof-mounted rain-out shelters to exclude natural precipitation, while its open sides allowed for natural ventilation. This greenhouse configuration prevented interference from rainfall and avoided excessive heat buildup, thereby ensuring that soil moisture was controlled solely by experimental treatments while maintaining microclimatic conditions suitable for seedling growth.

### 4.2. Tree Species Selection

Four broad-leaved tree species, including *K. paniculata*, *Q. variabilis*, *A. truncatum* and *P. davidiana*, with different growth rates and micro-site preference in natural forests in the shallow mountainous regions of northern China, were selected as study subjects. *P. davidiana*, with relatively limited lateral root differentiation, is a fast-growing species and develops in the early successional stage, and is often planted in infertile and sunny sites. *K. paniculata* grows more slowly than *P. davidiana* in the mid-to-late succession stage, and naturally distributes in fertile microsites, possessing a well-developed root system characterized by extensive lateral root branching. *Q. variabilis* grows more rapidly in nutrient- and water-rich environments, but exhibits slower growth rates in nutrient-poor habitats. Although its overall growth rate is slower than that of *K. paniculata*, it has relatively limited lateral root differentiation. *A. truncatum* is a typical slow-growing species in the late successional stage, characterized by a dense root system and strong tolerance to nutrient limitation.

### 4.3. Seedling Cultivation and Experiment Layout

Seeds of each species were collected from a single maternal tree within the Miaofengshan Experimental Forest Farm, the same locality as the experimental nursery, to minimize genetic variation within species and ensure that maternal trees had been exposed to uniform environmental conditions (i.e., consistent soil properties and climate). Due to interspecific differences in germination period [65], seeds were sown on staggered dates in seedling trays so that all seedlings reached a comparable developmental stage at the start of the experiment. To enhance germination synchrony, seeds were first mixed with moist river sand and subjected to cold stratification in the field as a pre-germination treatment [66]. Germinated seeds with uniform radicle lengths were then transplanted into seedling trays containing a 3:1 (*v*/*v*) peat–perlite mixture and maintained under routine irrigation. Uniform seedlings of four tree species were transplanted individually into 30 L PVC pots (30 cm diameter × 35 cm height) when they attained an average height of approximately 20 cm.

**Figure 8:**
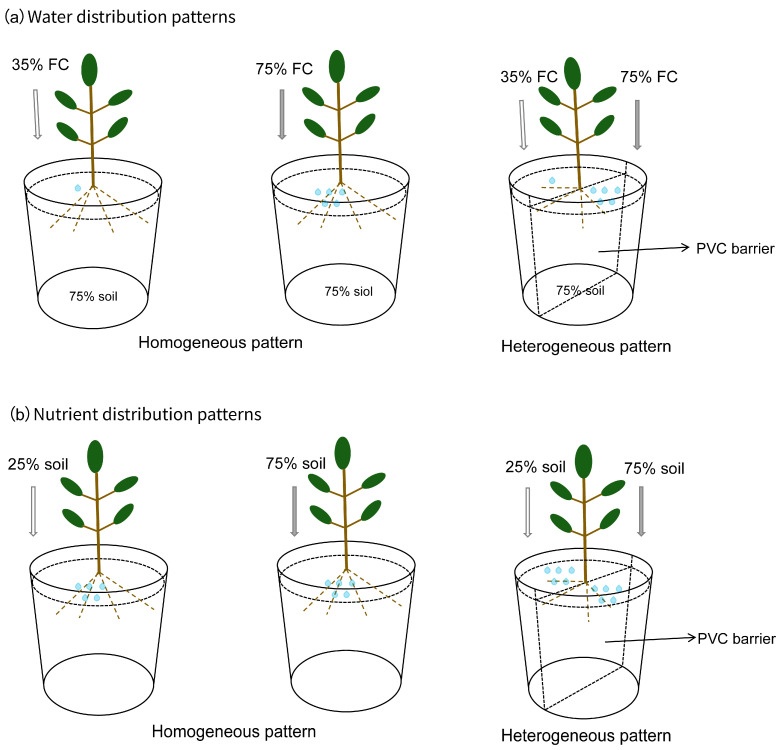
Layout of the pot experiment design showing (**a**) water and (**b**) nutrient distribution patterns.

A split-root experiment, a classical approach for evaluating root foraging behavior under soil resource heterogeneity [13,14], was conducted in early May 2024. The substrate was prepared by mixing sieved nursery soil (passed through a 4 mm mesh) with purified river sand at different volumetric ratios to mimic the textural gradients of natural shallow mountain soils. Both water and nutrient distributions were arranged in two patterns: homogeneous and heterogeneous (Figure 8). For heterogeneous treatments, a vertical PVC barrier divided the pot into two equal-volume compartments. A seedling was positioned astride the barrier, allowing fine roots to distribute evenly into both halves. For nutrient distribution, homogeneous patterns used a uniform soil mixture throughout the pot, with two levels: 75% soil (soil: sand = 75:25, *v*/*v*, nutrient-rich) or 25% soil (soil:sand = 25:75, *v*/*v*, nutrient stress). In heterogeneous patterns, a nutrient-rich patch (75% soil) and a nutrient-stressed patch (25% soil) were created in the two compartments of the same pot. Field capacity (FC) for each substrate mixture was determined beforehand using the cutting-ring method [67]; associated nutrient availability and physical properties are listed in Table S1. For water distribution, homogeneous patterns maintained uniform soil moisture throughout the pot at either a water-rich level (75% FC) or a water-stressed level (35% FC). Heterogeneous patterns imposed a water-rich patch (75% FC) and a water-stressed patch (35% FC) in separate compartments. All water-treatment pots used a uniform soil matrix corresponding to the nutrient-rich mixture (75% soil).

A total of 72 seedlings were grown under an experimental design comprising three treatments × three replicate units × four tree species × two distribution patterns. After transplantation, all seedlings were thoroughly watered and shaded for a one-week establishment period, during which substrate moisture was maintained near field capacity. Any seedlings that failed to establish were replaced. No exogenous fertilizers were applied during the experiment. Soil moisture was regulated automatically via a custom-built Intelligent Moisture Control System (Xinglian Zhitong Technology Co., Ltd., Beijing, China). Each pot was fitted with a drip emitter, and three moisture sensors were installed per treatment to monitor real-time water content. Irrigation was triggered based on the average sensor readings and adjusted daily via a mobile application to maintain the target water levels. All seedlings were subjected to nutrient and water treatments for a duration of five months (mid-May to mid-October).

### 4.4. Root Traits and Root Biomass Measurement

After one growing season under treatments, all seedling roots were carefully excised from shoots at the soil surface. Roots from each compartment were separated from the substrate. Fine and scattered root fragments in each soil layer were collected using a 4 mm mesh sieve, with care taken to minimize loss. All roots were immediately rinsed with distilled water, placed in moist paper towels, and transported to the laboratory.

Root orders were classified following Pregitzer et al. [68]. For root segments that became fragmented, order assignment was based on similarity in diameter and length, referencing Guo et al. [69]. Roots were scanned with a digital scanner (Epson Perfection V370 at a resolution of 300 dpi), and then root images were processed using WINRHIZO software (WINRHIZO Pro 2017a) to determine mean the average diameter (RD, mm), root length (RL, cm), root area (RA, cm^2^), root volume (RV, cm^3^), root tip number (RTN, n) of each root order. Subsequently, roots were oven-dried to constant mass and weighed on a balance with 0.0001 g precision to obtain dry mass. Using these measurements, root tissue density (RTD, dry root mass/root volume, g/cm^3^), specific root length (SRL, root length/dry root mass, cm/g), specific root area (SRA, root area/dry root mass, cm^2^/g), and root branching intensity (RBI, number of root tips/root length, n/cm) were calculated.

### 4.5. Statistical Analysis

Root foraging scale, including root biomass foraging scale (FS_RB_) and root length foraging scale (FS_RL_), was calculated as follows [3]:

(1) $${FS}_{RB}={Bio}_{T}+{Bio}_{CT}$$

(2) $${FS}_{RL}={RL}_{T}+{RL}_{CT}$$

Root foraging precision was assessed using root length foraging precision (FP_RL_), calculated as [4]:

(3) FPRL=100×RLCT−RLTRLT

Under homogeneous distribution patterns, subscripts T denotes the biomass and RL of fine roots from the stress treatment pots, and CT denotes those from the resource-rich pots; under heterogeneous patterns, T and CT refer to the root biomass or RL from the stress-side and resource-rich-side compartments within the same pot.

Analysis of variance (ANOVA) was used to analyze fine root foraging behavior. The full factorial ANOVA model included the main effects of species, root order, and treatment, as well as their two-way and three-way interactions. Results were compared by Tukey test (replicates, *n* = 3) at 0.05 level. When significant interactions were detected, simple effect analyzes were performed to further explore the nature of the interaction. Pearson correlation analysis was used to analyze the relationships between trait values in resource-rich patches and root foraging precision or scale at the resource patch level. The combined contributions of fine root morphological traits in high-resource patches to root foraging behavior variables, as well as the relationship between root foraging scale and precision, were evaluated using multivariate regression analysis to further explore the association between root foraging precision and root functional traits in a multidimensional trait space; principal component analysis (PCA) was performed across all four species.

All analyses were conducted using R 4.5.1.

## 5. Conclusions

This study addresses a critical gap in understanding root foraging behavior of four tree species in heterogeneous environments through controlled experimental manipulations. It not only provides a new insight into root adaptive strategies under water- and nutrient patch but also guides future research on plant responses to heterogeneous resource distributions. The results show that foraging strategies among tree species form a continuous spectrum along gradients of nutrient and water heterogeneity, rather than a dichotomous “precise foraging” versus “random walk” distinction. No clear trade-off was detected between foraging precision and scale. Furthermore, root traits drive nonlinear responses in foraging scale, yet root morphological traits cannot predict foraging precision, indicating that foraging precision is independent of traditional morphological trait spectra. These findings underscore the central role of root functional traits in heterogeneous resource use and the continuous nature of strategic differentiation among species, advancing our understanding of plant functional diversity in response to resource heterogeneity. Future studies should integrate mycorrhizal symbiosis and field-based validation to analyze how root–mycorrhizal interactions shape foraging strategies and to explore trade-off mechanisms of root functional traits across different resource types, thereby refining the theoretical framework for plant adaptation to heterogeneous environments.

## Data Availability

Data are available from the corresponding author on reasonable request.

## Supplementary Materials

Supplementary data of this study can be found in the article and/or Supplementary Information. The following supporting information can be downloaded at: https://www.mdpi.com/article/10.3390/plants15070998/s1, Table S1: The contents of nutrient elements and Field capacity for different soil percentage substrate; Table S2: ANOVA of the effects of tree species, root order, and patch treatment on root foraging scale; Table S3: ANOVA of the effects of tree species, root order, and patch heterogeneity on root foraging precision; Table S4: Pearson correlations of root foraging scale and precision with root traits in different resource; Table S5: Pearson correlation coefficient between PCA score and trait; Table S6: Pearson correlation coefficients between root foraging scale and precision; Figure S1: Root foraging scale under different water distribution patterns in 4 tree species across root orders.

## References

[1] Chen W., Koide R.T., Adams T.S., DeForest J.L., Cheng L., Eissenstat D.M. (2016). Root Morphology and Mycorrhizal Symbioses Together Shape Nutrient Foraging Strategies of Temperate Trees. Proc. Natl. Acad. Sci. USA.

[2] Skálová H., Jandová K., Balšánková T., Hadincová V., Krahulec F., Pecháčková S., Krak K., Herben T. (2023). Cations Make a Difference: Soil Nutrient Patches and Fine-scale Root Abundance of Individual Species in a Mountain Grassland. Funct. Ecol..

[3] Fan R., Tan L., Zheng M., Huang X., Liu X., Guo S. (2024). Assessment of Root Foraging Behaviour in Aralia Elata Subjected to Drought Stress under Different Light Spectra. Not. Bot. Horti Agrobot. Cluj-Napoca.

[4] Jiang Q., Jia L., Chen W., Zheng Z., Lin C., Zhu L., Wang X., Yao X., Tissue D., Robinson D. (2025). Complementary Foraging of Roots and Mycorrhizal Fungi Among Nutrient Patch Types in Four Subtropical Monospecific Broadleaved Tree Plantations. New Phytol..

[5] Träger S., Wilson S.D. (2017). Root Heterogeneity along an Arctic Elevational Gradient: The Importance of Resolution. Funct. Ecol..

[6] Lepik A., Abakumova M., Davison J., Zobel K., Semchenko M. (2021). Spatial Mapping of Root Systems Reveals Diverse Strategies of Soil Exploration and Resource Contest in Grassland Plants. J. Ecol..

[7] Wang G., Liu S., Fang Y., Shangguan Z. (2020). Adaptive Changes in Root Morphological Traits of Gramineae and Leguminosae Seedlings in the Ecological Restoration of the Semiarid Region of Northwest China. Land Degrad. Dev..

[8] Li H., Wang X., Brooker R.W., Rengel Z., Zhang F., Davies W.J., Shen J. (2019). Root Competition Resulting from Spatial Variation in Nutrient Distribution Elicits Decreasing Maize Yield at High Planting Density. Plant Soil.

[9] Wang P., Mou P., Hu L., Hu S. (2022). Effects of Nutrient Heterogeneity on Root Foraging and Plant Growth at the Individual and Community Level. J. Exp. Bot..

[10] Sun T., Ren R., Xing M., Duan Q., Zhao L., Yan W., Fang Y., Zhang S., Dong H., Wang M. (2024). Morphological and Physiological Plasticity of Catalpa Bungei Roots under Partial Root-Zone Drought as Affected by Nitrogen Forms. New For..

[11] Campbell B.D., Grime J.P., Mackey J.M.L. (1991). A Trade-off between Scale and Precision in Resource Foraging. Oecologia.

[12] Kembel S.W., De Kroon H., Cahill J.F., Mommer L. (2008). Improving the Scale and Precision of Hypotheses to Explain Root Foraging Ability. Ann. Bot..

[13] Wei H., Guo P., Zheng H., He X., Wang P., Ren Z., Zhai C. (2017). Micro-Scale Heterogeneity in Urban Forest Soils Affects Fine Root Foraging by Ornamental Seedlings of Buddhist Pine and Northeast Yew. Urban For. Urban Green..

[14] Zhou C., Cui W., Yuan T., Cheng H., Su Q., Wei H., Guo P. (2022). Root Foraging Behavior of Two Agronomical Herbs Subjected to Heterogeneous P Pattern and High Ca Stress. Agronomy.

[15] Giehl R.F.H., von Wiren N. (2014). Root Nutrient Foraging. Plant Physiol..

[16] Stiblíková P., Klimeš A., Cahill J.F., Koubek T., Weiser M. (2023). Interspecific Differences in Root Foraging Precision Cannot Be Directly Inferred from Species’ Mycorrhizal Status or Fine Root Economics. Oikos.

[17] Zhu L., Yao X., Chen W., Robinson D., Wang X., Chen T., Jiang Q., Jia L., Fan A., Wu D. (2023). Plastic Responses of Below-Ground Foraging Traits to Soil Phosphorus-Rich Patches Across 17 Coexisting AM Tree Species in a Subtropical Forest. J. Ecol..

[18] Liu Y., Von Wirén N. (2022). Integration of Nutrient and Water Availabilities via Auxin into the Root Developmental Program. Curr. Opin. Plant Biol..

[19] Wijesinghe D.K., John E.A., Beurskens S., Hutchings M.J. (2001). Root System Size and Precision in Nutrient Foraging: Responses to Spatial Pattern of Nutrient Supply in Six Herbaceous Species. J. Ecol..

[20] Chen B.M., Su J.Q., Liao H.X., Peng S.L. (2018). A Greater Foraging Scale, Not a Higher Foraging Precision, May Facilitate Invasion by Exotic Plants in Nutrient-Heterogeneous Conditions. Ann. Bot..

[21] Einsmann J.C., Jones R.H., Pu M., Mitchell A.J. (1999). Nutrient Foraging Traits in 10 Co-occurring Plant Species of Contrasting Life Forms. J. Ecol..

[22] Farley R.A., Fitter A.H. (1999). The Responses of Seven Co-occurring Woodland Herbaceous Perennials to Localized Nutrient-Rich Patches. J. Ecol..

[23] Blair B.C., Perfecto I. (2004). Successional Status and Root Foraging for Phosphorus in Seven Tropical Tree Species. Can. J. For. Res..

[24] Grime J.P. (2007). The Scale-Precision Trade-off in Spacial Resource Foraging by Plants: Restoring Perspective. Ann. Bot..

[25] Reyes M.F., Aguiar M.R. (2017). Root Proliferation Strategies and Exploration of Soil Patchiness in Arid Communities. Austral Ecol..

[26] Liu B., Li L., Rengel Z., Tian J., Li H., Lu M. (2019). Roots and Arbuscular Mycorrhizal Fungi Are Independent in Nutrient Foraging across Subtropical Tree Species. Plant Soil.

[27] Yang Z., Zhou B., Ge X., Cao Y., Brunner I., Shi J., Li M.-H. (2021). Species-Specific Responses of Root Morphology of Three Co-Existing Tree Species to Nutrient Patches Reflect Their Root Foraging Strategies. Front. Plant Sci..

[28] Eissenstat D.M., Kucharski J.M., Zadworny M., Adams T.S., Koide R.T. (2015). Linking Root Traits to Nutrient Foraging in Arbuscular Mycorrhizal Trees in a Temperate Forest. New Phytol..

[29] Liu B., Li H., Zhu B., Koide R.T., Eissenstat D.M., Guo D. (2015). Complementarity in Nutrient Foraging Strategies of Absorptive Fine Roots and Arbuscular Mycorrhizal Fungi across 14 Coexisting Subtropical Tree Species. New Phytol..

[30] Chen W., Koide R.T., Eissenstat D.M. (2018). Nutrient Foraging by Mycorrhizas: From Species Functional Traits to Ecosystem Processes. Funct. Ecol..

[31] Kramer-Walter K.R., Bellingham P.J., Millar T.R., Smissen R.D., Richardson S.J., Laughlin D.C. (2016). Root Traits Are Multidimensional: Specific Root Length Is Independent from Root Tissue Density and the Plant Economic Spectrum. J. Ecol..

[32] Ceulemans T., Bodé S., Bollyn J., Harpole S., Coorevits K., Peeters G., Van Acker K., Smolders E., Boeckx P., Honnay O. (2017). Phosphorus Resource Partitioning Shapes Phosphorus Acquisition and Plant Species Abundance in Grasslands. Nat. Plants.

[33] Wang J., Xu T., Wang Y., Li G., Abdullah I., Zhong Z., Liu J., Zhu W., Wang L., Wang D. (2021). A Meta-analysis of Effects of Physiological Integration in Clonal Plants under Homogeneous vs. Heterogeneous Environments. Funct. Ecol..

[34] Li H., Zhang D., Wang X., Li H., Rengel Z., Shen J. (2019). Competition between Zea Mays Genotypes with Different Root Morphological and Physiological Traits Is Dependent on Phosphorus Forms and Supply Patterns. Plant Soil.

[35] Xia H., Zhang T., Li X., He T., Wang X., Zhang J., Zhang K. (2023). Effects of Drought and Nutrient Deficiencies on the Allocation of Recently Fixed Carbon in a Plant–Soil–Microbe System. Tree Physiol..

[36] Wei L.D. (2021). Adaptation of Root Functional Traits of Landscape Tree Species to Dry and Barren Site in Stony Mountainous Area in Beijing. Master’s Thesis.

[37] Shen J., Li C., Mi G., Li L., Yuan L., Jiang R., Zhang F. (2013). Maximizing Root/Rhizosphere Efficiency to Improve Crop Productivity and Nutrient Use Efficiency in Intensive Agriculture of China. J. Exp. Bot..

[38] Robinson D. (1994). The Responses of Plants to Non-uniform Supplies of Nutrients. New Phytol..

[39] Liu B., Han F., Xing K., Zhang A., Rengel Z. (2021). The Response of Plants and Mycorrhizal Fungi to Nutritionally-Heterogeneous Environments Are Regulated by Nutrient Types and Plant Functional Groups. Front. Plant Sci..

[40] Hodge A. (2004). The Plastic Plant: Root Responses to Heterogeneous Supplies of Nutrients. New Phytol..

[41] Li H., Ma Q., Li H., Zhang F., Rengel Z., Shen J. (2014). Root Morphological Responses to Localized Nutrient Supply Differ among Crop Species with Contrasting Root Traits. Plant Soil.

[42] Robinson D., BassiriRad H. (2005). Integrated Root Responses to Variations in Nutrient Supply. Nutrient Acquisition by Plants: An Ecological Perspective.

[43] Ruan L., Cheng H., Ludewig U., Li J., Chang S.X. (2022). Root Foraging Strategy Improves the Adaptability of Tea Plants (*Camellia sinensis* L.) to Soil Potassium Heterogeneity. IJMS.

[44] Chen W., Koide R.T., Eissenstat D.M. (2018). Root Morphology and Mycorrhizal Type Strongly Influence Root Production in Nutrient Hot Spots of Mixed Forests. J. Ecol..

[45] Nasr Esfahani M., Sonnewald U. (2024). Unlocking Dynamic Root Phenotypes for Simultaneous Enhancement of Water and Phosphorus Uptake. Plant Physiol. Biochem..

[46] Pierik R., Testerink C. (2014). The Art of Being Flexible: How to Escape from Shade, Salt, and Drought. Plant Physiol..

[47] Yang J., Zhou Z., Qi W., Gao X., Wang Y., Wang X., Yi X., Ma M., Wu S. (2025). Phenotypic Plasticity and Integration Synergistically Enhance Plant Adaptability to Flooding and Nitrogen Stresses. Plant Soil..

[48] Ploschuk R.A., Miralles D.J., Colmer T.D., Ploschuk E.L., Striker G.G. (2020). Corrigendum: Waterlogging of Winter Crops at Early and Late Stages: Impacts on Leaf Physiology, Growth and Yield. Front. Plant Sci..

[49] Mommer L., Visser E.J.W., Van Ruijven J., De Caluwe H., Pierik R., De Kroon H. (2011). Contrasting Root Behaviour in Two Grass Species: A Test of Functionality in Dynamic Heterogeneous Conditions. Plant Soil.

[50] Dekroon H., Mommer L. (2006). Root Foraging Theory Put to the Test. Trends Ecol. Evol..

[51] Forde B.G., Walch-Liu P. (2009). Nitrate and Glutamate as Environmental Cues for Behavioural Responses in Plant Roots. Plant Cell Environ..

[52] Wang P., Shu M., Mou P., Weiner J. (2018). Fine Root Responses to Temporal Nutrient Heterogeneity and Competition in Seedlings of Two Tree Species with Different Rooting Strategies. Ecol. Evol..

[53] Adams T.S., McCormack M.L., Eissenstat D.M. (2013). Foraging Strategies in Trees of Different Root Morphology: The Role of Root Lifespan. Tree Physiol..

[54] Luke McCormack M., Adams T.S., Smithwick E.A.H., Eissenstat D.M. (2012). Predicting Fine Root Lifespan from Plant Functional Traits in Temperate Trees. New Phytol..

[55] Li D., Nan H., Liang J., Cheng X., Zhao C., Yin H., Yin C., Liu Q. (2017). Responses of Nutrient Capture and Fine Root Morphology of Subalpine Coniferous Tree Picea Asperata to Nutrient Heterogeneity and Competition. PLoS ONE.

[56] Semchenko M., Lepik A., Abakumova M., Zobel K. (2018). Different Sets of Belowground Traits Predict the Ability of Plant Species to Suppress and Tolerate Their Competitors. Plant Soil.

[57] Freschet G.T., Violle C., Bourget M.Y., Scherer-Lorenzen M., Fort F. (2018). Allocation, Morphology, Physiology, Architecture: The Multiple Facets of Plant Above- and Below-Ground Responses to Resource Stress. New Phytol..

[58] de la Riva E.G., Prieto I., Marañón T., Pérez-Ramos I.M., Olmo M., Villar R. (2021). Root Economics Spectrum and Construction Costs in Mediterranean Woody Plants: The Role of Symbiotic Associations and the Environment. J. Ecol..

[59] Augusto L., Borelle R., Boča A., Bon L., Orazio C., Arias-González A., Bakker M.R., Gartzia-Bengoetxea N., Auge H., Bernier F. (2025). Widespread Slow Growth of Acquisitive Tree Species. Nature.

[60] Huang X., Lu Z., Li F., Deng Y., Wan F., Wang Q., Folega F., Wang J., Guo Z. (2024). Evolution History Dominantly Regulates Fine Root Lifespan in Tree Species Across the World. For. Ecosyst..

[61] Liang S., Guo H., McCormack M.L., Qian Z., Huang K., Yang Y., Xi M., Qi X., Ou X., Liu Y. (2023). Positioning Absorptive Root Respiration in the Root Economics Space across Woody and Herbaceous Species. J. Ecol..

[62] Hou J., McCormack M.L., Reich P.B., Sun T., Phillips R.P., Lambers H., Chen H.Y.H., Ding Y., Comas L.H., Valverde-Barrantes O.J. (2024). Linking Fine Root Lifespan to Root Chemical and Morphological Traits—A Global Analysis. Proc. Natl. Acad. Sci. USA.

[63] Pregitzer K.S., Laskowski M.J., Burton A.J., Lessard V.C., Zak D.R. (1998). Variation in Sugar Maple Root Respiration with Root Diameter and Soil Depth. Tree Physiol..

[64] Van Der Bom F.J.T., Williams A., Bell M.J. (2020). Root Architecture for Improved Resource Capture: Trade-Offs in Complex Environments. J. Exp. Bot..

[65] Liu Y.J., Oduor A.M.O., Dai Z., Gao F., Li J., Zhang X., Yu F. (2021). Suppression of a Plant Hormone Gibberellin Reduces Growth of Invasive Plants More than Native Plants. Oikos.

[66] Li J.H. (2020). Mechanical Responses of Leaf and Root Morphological and Physiological Traits of *Cotinus coggygria* Seedlings to Drought and Barren Soil. Ph.D. Thesis.

[67] Yu M., Zhang B.J., Wang Z.J., Yu F.Z., Zhao X.H., Yang J., Li P., Fan D.Y., Xu C.Y. (2024). Relationship of Functional Traits and Site Conditions with NO_3~-Uptake Capacity of Tree Root. J. Beijing For. Univ..

[68] Pregitzer K.S., DeForest J.L., Burton A.J., Allen M.F., Ruess R.W., Hendrick R.L. (2002). Fine Root Architecture of Nine North American Trees. Ecol. Monogr..

[69] Guo D.L., Mitchell R.J., Hendricks J.J. (2004). Fine Root Branch Orders Respond Differentially to Carbon Source-Sink Manipulations in a Longleaf Pine Forest. Oecologia.

