# Supplementary material for: Morphological Traits Shape Foraging Scale but Not Precision: Divergent Responses of Four Tree Species to Water and Nutrient Heterogeneity
Source: Plants (Basel). 2026 Mar 24;15(7):998. doi: 10.3390/plants15070998 (PMC13075164; doi:10.3390/plants15070998)
Supplement: Supplementary file 1 [file plants-15-00998-s001.zip › plants-4145974-supplementary.pdf]

Morphological Traits Shape Foraging Scale but Not Precision: Divergent Responses of Four Tree Species to Water and Nutrient Heterogeneity

Liuduan Wei [1], Tianxin Dong [1], Liufeng Lan [2], Jian Lin [3], Xianwen Li [1], Miao Yu [1,*] and Chengyang Xu [1,*]

[1] Research Center for Urban Forestry at Beijing Forestry University, Key Laboratory for Silviculture and Forest Ecosystem of State Forestry and Grassland Administration, the College of forestry, Beijing Forestry University, Beijing 100083, P.R. China
[2] Guangxi State-owned Huangmian Forest Farm, Liuzhou 545618, P.R. China
[3] Nanning Arboretum, Guangxi Zhuang Autonomous Region, Nanning 530033, P.R. China
[*] Correspondence: cyxu@bjfu.edu.cn (Chengyang Xu), yumiao2020@bjfu.edu.cn (Miao Yu).

Table S1 The contents of nutrient elements and Field capacity for different soil percentage substrate

Soil percentage (%)	Nitrogen (g/kg)	Phosphorus (g/kg)	Available phosphorus (mg/kg)	Available nitrogen (mg/kg)	Field capacity（%）
25	1.28	0.62	8.54	9.17	14.67
75	1.37	0.67	12.31	22.92	17.66

Table S2 ANOVA of the effects of tree species, root order, and patch treatment on root foraging scale

	FS_{RB}			FS_{RL}		
	df	*F*	*P*	*df*	*F*	*P*
Species	3	146.59	<0.001	3	127.19	<0.001
Root order	5	248.56	<0.001	5	413.75	<0.001
Treatment	5	4900.49	<0.001	5	7354.21	<0.001
Species×Root order	15	3.4343	<0.001	15	2.207	0.0063
Species×Treatment	15	35.04	<0.001	15	23.48	<0.001
Root order×Treatment	25	28.14	<0.001	25	61.47	<0.001
Species×Root Order×Treatment	75	1.301	0.065	75	1.958	<0.001
Residuals	294			294		

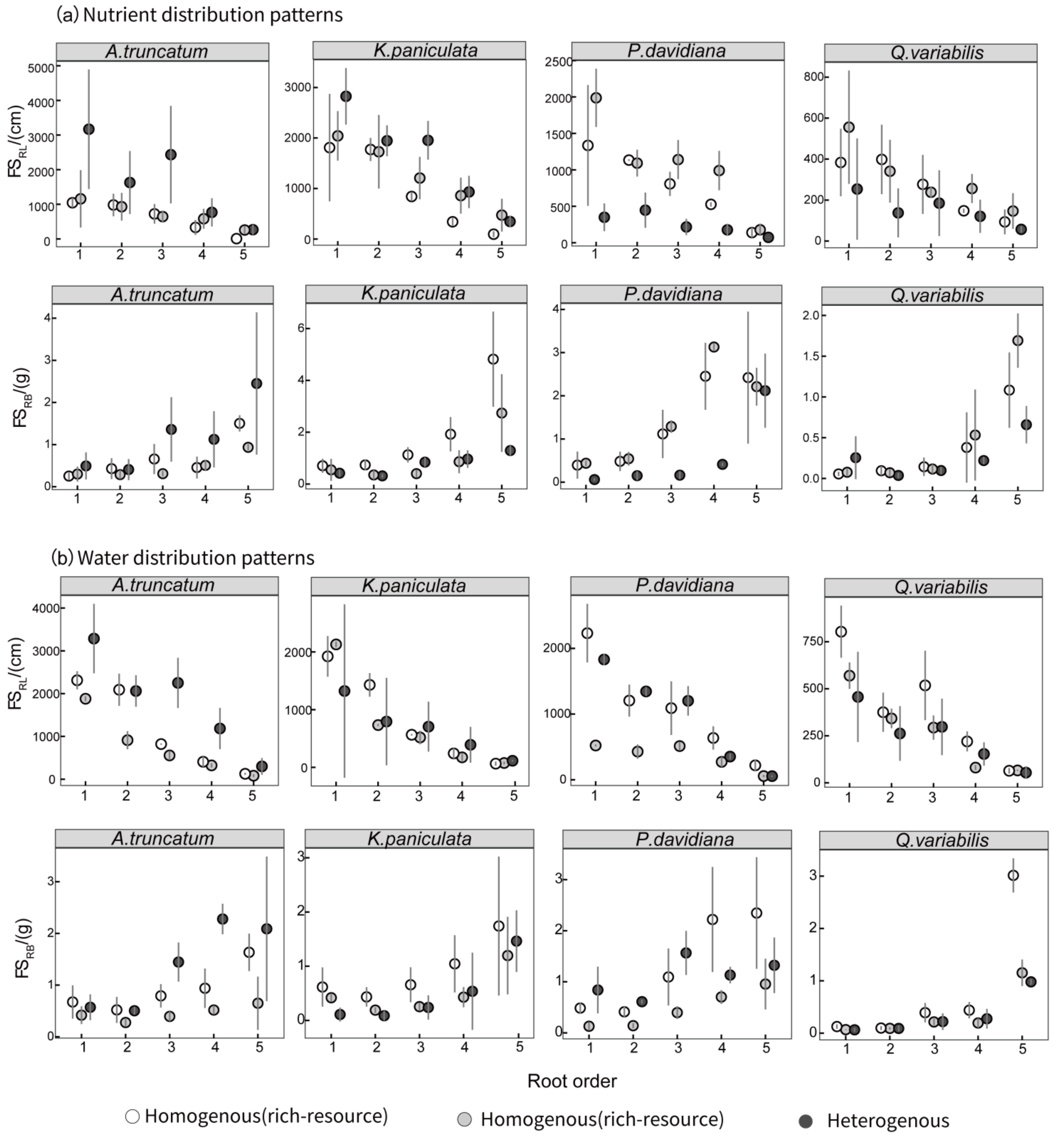

Figure S1 Root foraging scale under different water distribution patterns in 4 tree species across root orders.

Table S3 ANOVA of the effects of tree species, root order, and patch heterogeneity on root foraging precision

	Water distribution pattern			Nutrient distribution pattern		
	df	*F*	*P*	*df*	*F*	*P*
Hetero or Homo pattern	1	3.69	0.057	1	0.47	0.495
Root order	4	1.05	0.393	4	1.52	0.191
Species	3	10.81	<0.001	3	13.95	<0.001
Pattern×Root order	5	0.65	0.660	5	0.47	0.755
Patterns×Species	3	9.46	<0.001	3	8.50	<0.001
Pattern×Root order×Species	15	1.27	0.237	15	1.28	0.241
Residuals	96			96		

Table S4 Pearson correlations of root foraging scale and precision with root traits in different resource patches

	Traits	RL	RD	RTN	SRL	SRA	RTD	RBI
Nutrient	FS_{RB}	0.030	0.382*	-0.095	-0.570**	-0.429**	0.207	-0.314
	FS_{RL}	0.904**	-0.301	0.832**	0.319*	0.361*	0.087	0.021
	FP_{RL}	-0.214	0.259	-1.177	-0.067	-0.140	-0.030	0.150
Water	FS_{RB}	-0.012	0.647**	-0.057	-0.432**	-0.314*	-0.073	-0.308*
	FS_{RL}	0.934**	-0.257	0.806**	0.331*	0.502**	-0.145	0.164
	FP_{RL}	0.046	-0.127	0.029	-0.010	0.021	0.072	0.071

*, ** and *** indicate significance at $P < 0.05$, $P < 0.01$ and $P < 0.001$ level, respectively.

Table S5 Pearson correlation coefficient between PCA score and trait

	FP_{RL}	RD	SRL	SRA	RTD	RBI
PC1	0.10	**-0.58**	**0.94**	0.73	-0.06	0.77
PC2	-0.39	0.57	0.20	**0.62**	**-0.77**	-0.33
PC3	**-0.90**	-0.14	0.03	0.00	0.36	0.00

Table S6 Pearson correlation coefficients between root foraging scale and precision

	FS	FP_{RL}
Water distribution pattern	FS_{RB}	-0.011
	FS_{RL}	-0.037

Nutrient distribution pattern	FS_{RB}	0.168
	FS_{RL}	0.296
